# 

**Published:** 1886-06

**Authors:** 


### CONTENTS.

### PROCEEDINGS.

Louisiana State Dental Society.......... 267
Northern Ohio Dental Association.......... 291

### SELECTIONS.

Faith Healing.......... 303
The Finger Nails in Disease.......... 306
Manicure.......... 308
Anæsthetics.......... 309
Injections of Osmium Acid in Neuralgia.......... 310
A Rational Conclusion.......... 311
Beware of Bogus Dentists.......... 312

SALMAGUNDI.......... 313

### EDITORIAL.

International Congress..........316–318
Editorial Change.......... 319
National Dental Association.......... 320
Board of Examiners .......... 320

